# Supplementary material for: Heparin-based hydrogel scaffolding alters the transcriptomic profile and increases the chemoresistance of MDA-MB-231 triple-negative breast cancer cells
Source: Biomater Sci. 2020 Feb 13;8(10):2786–96. doi: 10.1039/c9bm01481k (PMC7497406; doi:10.1039/c9bm01481k)
Supplement: Supplementary file 2 [file BM-008-C9BM01481K-s002.zip › Supplementary File 4/HP-B hydrogel + EGF (s)vControl/Pathways/my_analysis.Gsea.1545201170987/HALLMARK_MYC_TARGETS_V1.html]

Details for gene set HALLMARK\_MYC\_TARGETS\_V1[GSEA]

|  || Dataset | expr.class.cls#HYDEGF\_versus\_CONTROL.class.cls#HYDEGF\_versus\_CONTROL\_repos |
| Phenotype | class.cls#HYDEGF\_versus\_CONTROL\_repos |
| Upregulated in class | CONTROL |
| GeneSet | HALLMARK\_MYC\_TARGETS\_V1 |
| Enrichment Score (ES) | -0.40089256 |
| Normalized Enrichment Score (NES) | -1.9179155 |
| Nominal p-value | 0.0 |
| FDR q-value | 6.6197186E-4 |
| FWER p-Value | 0.005 |
Table: GSEA Results Summary

  

Fig 1: Enrichment plot: HALLMARK\_MYC\_TARGETS\_V1      
 Profile of the Running ES Score & Positions of GeneSet Members on the Rank Ordered List

  

| PROBE | DESCRIPTION (from dataset) | GENE SYMBOL | GENE\_TITLE | RANK IN GENE LIST | RANK METRIC SCORE | RUNNING ES | CORE ENRICHMENT || 1 | CAD | na |  |  | 576 | 2.038 | -0.0169 | No |
| 2 | MCM2 | na |  |  | 778 | 1.847 | -0.0152 | No |
| 3 | NOP56 | na |  |  | 837 | 1.790 | -0.0065 | No |
| 4 | EIF3B | na |  |  | 839 | 1.788 | 0.0053 | No |
| 5 | EXOSC7 | na |  |  | 975 | 1.702 | 0.0094 | No |
| 6 | MCM7 | na |  |  | 1322 | 1.505 | 0.0011 | No |
| 7 | RNPS1 | na |  |  | 1371 | 1.483 | 0.0084 | No |
| 8 | PABPC4 | na |  |  | 1937 | 1.282 | -0.0129 | No |
| 9 | CDC45 | na |  |  | 2137 | 1.228 | -0.0153 | No |
| 10 | RUVBL2 | na |  |  | 2219 | 1.205 | -0.0116 | No |
| 11 | TRIM28 | na |  |  | 2284 | 1.186 | -0.0071 | No |
| 12 | EIF4A1 | na |  |  | 2683 | 1.084 | -0.0209 | No |
| 13 | MCM5 | na |  |  | 2694 | 1.082 | -0.0143 | No |
| 14 | TFDP1 | na |  |  | 2728 | 1.072 | -0.0090 | No |
| 15 | PRPF31 | na |  |  | 2749 | 1.065 | -0.0030 | No |
| 16 | MCM4 | na |  |  | 3125 | 0.976 | -0.0163 | No |
| 17 | RRP9 | na |  |  | 3207 | 0.957 | -0.0142 | No |
| 18 | CDK2 | na |  |  | 3627 | 0.880 | -0.0305 | No |
| 19 | SRM | na |  |  | 3681 | 0.869 | -0.0275 | No |
| 20 | IFRD1 | na |  |  | 3686 | 0.868 | -0.0220 | No |
| 21 | ODC1 | na |  |  | 3955 | 0.820 | -0.0307 | No |
| 22 | AIMP2 | na |  |  | 4042 | 0.805 | -0.0299 | No |
| 23 | SRSF7 | na |  |  | 4115 | 0.795 | -0.0284 | No |
| 24 | TRA2B | na |  |  | 4229 | 0.777 | -0.0293 | No |
| 25 | MYC | na |  |  | 4314 | 0.764 | -0.0286 | No |
| 26 | CDC20 | na |  |  | 4567 | 0.724 | -0.0371 | No |
| 27 | U2AF1 | na |  |  | 5425 | 0.587 | -0.0783 | No |
| 28 | IARS | na |  |  | 5493 | 0.577 | -0.0781 | No |
| 29 | CUL1 | na |  |  | 5551 | 0.570 | -0.0773 | No |
| 30 | NOLC1 | na |  |  | 5664 | 0.551 | -0.0795 | No |
| 31 | TYMS | na |  |  | 5792 | 0.533 | -0.0827 | No |
| 32 | SF3B3 | na |  |  | 5849 | 0.525 | -0.0822 | No |
| 33 | CLNS1A | na |  |  | 6532 | 0.433 | -0.1152 | No |
| 34 | CYC1 | na |  |  | 6701 | 0.411 | -0.1213 | No |
| 35 | EIF3D | na |  |  | 6748 | 0.404 | -0.1211 | No |
| 36 | SRSF3 | na |  |  | 6771 | 0.399 | -0.1196 | No |
| 37 | NOP16 | na |  |  | 6819 | 0.395 | -0.1195 | No |
| 38 | RANBP1 | na |  |  | 6835 | 0.393 | -0.1177 | No |
| 39 | DDX21 | na |  |  | 6980 | 0.373 | -0.1228 | No |
| 40 | FAM120A | na |  |  | 7415 | 0.315 | -0.1435 | No |
| 41 | SRSF1 | na |  |  | 7695 | 0.280 | -0.1564 | No |
| 42 | POLD2 | na |  |  | 7915 | 0.254 | -0.1662 | No |
| 43 | DHX15 | na |  |  | 8336 | 0.204 | -0.1870 | No |
| 44 | ORC2 | na |  |  | 8584 | 0.176 | -0.1988 | No |
| 45 | PPM1G | na |  |  | 8782 | 0.153 | -0.2082 | No |
| 46 | GNL3 | na |  |  | 8823 | 0.144 | -0.2093 | No |
| 47 | RPS2 | na |  |  | 8885 | 0.137 | -0.2116 | No |
| 48 | STARD7 | na |  |  | 9320 | 0.085 | -0.2339 | No |
| 49 | KPNB1 | na |  |  | 9341 | 0.083 | -0.2344 | No |
| 50 | SF3A1 | na |  |  | 9562 | 0.067 | -0.2455 | No |
| 51 | GOT2 | na |  |  | 9565 | 0.067 | -0.2452 | No |
| 52 | G3BP1 | na |  |  | 9872 | 0.026 | -0.2611 | No |
| 53 | PSMA7 | na |  |  | 10041 | 0.014 | -0.2698 | No |
| 54 | USP1 | na |  |  | 10213 | -0.009 | -0.2788 | No |
| 55 | MCM6 | na |  |  | 10326 | -0.026 | -0.2845 | No |
| 56 | KARS | na |  |  | 10741 | -0.071 | -0.3058 | No |
| 57 | COPS5 | na |  |  | 10746 | -0.072 | -0.3055 | No |
| 58 | MRPL9 | na |  |  | 10772 | -0.076 | -0.3064 | No |
| 59 | BUB3 | na |  |  | 10780 | -0.077 | -0.3062 | No |
| 60 | SNRPD1 | na |  |  | 11061 | -0.116 | -0.3202 | No |
| 61 | CCNA2 | na |  |  | 11402 | -0.146 | -0.3371 | No |
| 62 | TUFM | na |  |  | 11463 | -0.157 | -0.3392 | No |
| 63 | DDX18 | na |  |  | 11492 | -0.163 | -0.3396 | No |
| 64 | CSTF2 | na |  |  | 11717 | -0.189 | -0.3502 | No |
| 65 | NCBP1 | na |  |  | 11861 | -0.213 | -0.3563 | No |
| 66 | NME1 | na |  |  | 12003 | -0.217 | -0.3623 | No |
| 67 | DEK | na |  |  | 12009 | -0.218 | -0.3611 | No |
| 68 | XPOT | na |  |  | 12013 | -0.218 | -0.3598 | No |
| 69 | ABCE1 | na |  |  | 12033 | -0.222 | -0.3593 | No |
| 70 | C1QBP | na |  |  | 12154 | -0.237 | -0.3641 | No |
| 71 | CCT5 | na |  |  | 12176 | -0.240 | -0.3636 | No |
| 72 | XRCC6 | na |  |  | 12225 | -0.249 | -0.3645 | No |
| 73 | CDK4 | na |  |  | 12236 | -0.251 | -0.3634 | No |
| 74 | PSMC6 | na |  |  | 12333 | -0.266 | -0.3667 | No |
| 75 | POLE3 | na |  |  | 12483 | -0.284 | -0.3726 | No |
| 76 | EIF4H | na |  |  | 12487 | -0.284 | -0.3709 | No |
| 77 | H2AFZ | na |  |  | 12572 | -0.292 | -0.3734 | No |
| 78 | SNRPA1 | na |  |  | 12646 | -0.305 | -0.3752 | No |
| 79 | UBA2 | na |  |  | 12736 | -0.320 | -0.3778 | No |
| 80 | TARDBP | na |  |  | 12851 | -0.335 | -0.3816 | No |
| 81 | XPO1 | na |  |  | 13014 | -0.343 | -0.3878 | No |
| 82 | FBL | na |  |  | 13035 | -0.346 | -0.3866 | No |
| 83 | PCBP1 | na |  |  | 13203 | -0.371 | -0.3930 | No |
| 84 | GSPT1 | na |  |  | 13252 | -0.378 | -0.3930 | No |
| 85 | CCT2 | na |  |  | 13364 | -0.396 | -0.3962 | No |
| 86 | PA2G4 | na |  |  | 13412 | -0.405 | -0.3960 | No |
| 87 | VBP1 | na |  |  | 13425 | -0.407 | -0.3940 | No |
| 88 | TXNL4A | na |  |  | 13469 | -0.409 | -0.3935 | No |
| 89 | PSMC4 | na |  |  | 13485 | -0.411 | -0.3916 | No |
| 90 | NHP2 | na |  |  | 13491 | -0.412 | -0.3892 | No |
| 91 | SNRPA | na |  |  | 13518 | -0.416 | -0.3878 | No |
| 92 | RRM1 | na |  |  | 13525 | -0.417 | -0.3853 | No |
| 93 | HDGF | na |  |  | 13545 | -0.420 | -0.3836 | No |
| 94 | RFC4 | na |  |  | 13625 | -0.429 | -0.3849 | No |
| 95 | CBX3 | na |  |  | 13745 | -0.448 | -0.3882 | No |
| 96 | PHB2 | na |  |  | 13845 | -0.465 | -0.3903 | No |
| 97 | RSL1D1 | na |  |  | 13862 | -0.468 | -0.3881 | No |
| 98 | EPRS | na |  |  | 13907 | -0.472 | -0.3873 | No |
| 99 | IMPDH2 | na |  |  | 14016 | -0.491 | -0.3897 | No |
| 100 | SRPK1 | na |  |  | 14207 | -0.505 | -0.3964 | No |
| 101 | ILF2 | na |  |  | 14241 | -0.511 | -0.3948 | No |
| 102 | VDAC3 | na |  |  | 14280 | -0.516 | -0.3934 | No |
| 103 | RAN | na |  |  | 14371 | -0.532 | -0.3946 | No |
| 104 | CNBP | na |  |  | 14420 | -0.541 | -0.3935 | No |
| 105 | HSPD1 | na |  |  | 14479 | -0.552 | -0.3929 | No |
| 106 | HNRNPU | na |  |  | 14621 | -0.576 | -0.3966 | No |
| 107 | SET | na |  |  | 14647 | -0.580 | -0.3941 | No |
| 108 | SRSF2 | na |  |  | 14778 | -0.601 | -0.3969 | Yes |
| 109 | CCT3 | na |  |  | 14779 | -0.601 | -0.3930 | Yes |
| 110 | PRPS2 | na |  |  | 14795 | -0.602 | -0.3898 | Yes |
| 111 | ETF1 | na |  |  | 14811 | -0.605 | -0.3866 | Yes |
| 112 | TCP1 | na |  |  | 14889 | -0.617 | -0.3866 | Yes |
| 113 | LSM7 | na |  |  | 14891 | -0.618 | -0.3825 | Yes |
| 114 | PSMD3 | na |  |  | 14918 | -0.621 | -0.3798 | Yes |
| 115 | PSMA1 | na |  |  | 14939 | -0.626 | -0.3767 | Yes |
| 116 | PCNA | na |  |  | 14963 | -0.629 | -0.3738 | Yes |
| 117 | MAD2L1 | na |  |  | 15013 | -0.635 | -0.3722 | Yes |
| 118 | MRPL23 | na |  |  | 15059 | -0.644 | -0.3703 | Yes |
| 119 | SERBP1 | na |  |  | 15127 | -0.658 | -0.3695 | Yes |
| 120 | SLC25A3 | na |  |  | 15128 | -0.659 | -0.3651 | Yes |
| 121 | RPS3 | na |  |  | 15164 | -0.662 | -0.3626 | Yes |
| 122 | HSPE1 | na |  |  | 15347 | -0.696 | -0.3676 | Yes |
| 123 | PSMA2 | na |  |  | 15449 | -0.711 | -0.3682 | Yes |
| 124 | PSMD14 | na |  |  | 15549 | -0.732 | -0.3686 | Yes |
| 125 | RAD23B | na |  |  | 15689 | -0.759 | -0.3709 | Yes |
| 126 | PRDX4 | na |  |  | 15690 | -0.760 | -0.3659 | Yes |
| 127 | SNRPG | na |  |  | 15705 | -0.764 | -0.3616 | Yes |
| 128 | EIF1AX | na |  |  | 15777 | -0.780 | -0.3602 | Yes |
| 129 | RPL6 | na |  |  | 15916 | -0.804 | -0.3621 | Yes |
| 130 | PHB | na |  |  | 15933 | -0.807 | -0.3576 | Yes |
| 131 | HDDC2 | na |  |  | 15946 | -0.809 | -0.3529 | Yes |
| 132 | SMARCC1 | na |  |  | 15991 | -0.816 | -0.3499 | Yes |
| 133 | SYNCRIP | na |  |  | 16017 | -0.824 | -0.3457 | Yes |
| 134 | CCT7 | na |  |  | 16067 | -0.837 | -0.3428 | Yes |
| 135 | EIF3J | na |  |  | 16142 | -0.855 | -0.3411 | Yes |
| 136 | PSMD7 | na |  |  | 16195 | -0.868 | -0.3381 | Yes |
| 137 | PTGES3 | na |  |  | 16323 | -0.904 | -0.3388 | Yes |
| 138 | EIF2S1 | na |  |  | 16348 | -0.909 | -0.3340 | Yes |
| 139 | HNRNPA2B1 | na |  |  | 16367 | -0.915 | -0.3290 | Yes |
| 140 | NAP1L1 | na |  |  | 16373 | -0.918 | -0.3232 | Yes |
| 141 | PSMA6 | na |  |  | 16408 | -0.925 | -0.3189 | Yes |
| 142 | SSBP1 | na |  |  | 16444 | -0.935 | -0.3145 | Yes |
| 143 | KPNA2 | na |  |  | 16498 | -0.947 | -0.3111 | Yes |
| 144 | RPS6 | na |  |  | 16538 | -0.954 | -0.3068 | Yes |
| 145 | SSB | na |  |  | 16581 | -0.965 | -0.3027 | Yes |
| 146 | SNRPD2 | na |  |  | 16688 | -0.997 | -0.3017 | Yes |
| 147 | RPS5 | na |  |  | 16694 | -0.999 | -0.2954 | Yes |
| 148 | HNRNPC | na |  |  | 16722 | -1.005 | -0.2901 | Yes |
| 149 | PSMB2 | na |  |  | 16760 | -1.014 | -0.2854 | Yes |
| 150 | RPL18 | na |  |  | 16788 | -1.023 | -0.2801 | Yes |
| 151 | CCT4 | na |  |  | 16841 | -1.040 | -0.2759 | Yes |
| 152 | PSMA4 | na |  |  | 16892 | -1.051 | -0.2716 | Yes |
| 153 | ACP1 | na |  |  | 16940 | -1.069 | -0.2671 | Yes |
| 154 | PSMD1 | na |  |  | 16955 | -1.073 | -0.2607 | Yes |
| 155 | GLO1 | na |  |  | 16971 | -1.078 | -0.2544 | Yes |
| 156 | EIF2S2 | na |  |  | 17005 | -1.091 | -0.2489 | Yes |
| 157 | NCBP2 | na |  |  | 17024 | -1.096 | -0.2427 | Yes |
| 158 | HNRNPA1 | na |  |  | 17030 | -1.098 | -0.2357 | Yes |
| 159 | MRPS18B | na |  |  | 17168 | -1.134 | -0.2354 | Yes |
| 160 | PWP1 | na |  |  | 17246 | -1.159 | -0.2318 | Yes |
| 161 | EIF4E | na |  |  | 17315 | -1.190 | -0.2275 | Yes |
| 162 | HNRNPR | na |  |  | 17326 | -1.192 | -0.2202 | Yes |
| 163 | PSMD8 | na |  |  | 17329 | -1.193 | -0.2124 | Yes |
| 164 | PSMB3 | na |  |  | 17344 | -1.196 | -0.2053 | Yes |
| 165 | DUT | na |  |  | 17427 | -1.226 | -0.2015 | Yes |
| 166 | HNRNPA3 | na |  |  | 17520 | -1.267 | -0.1980 | Yes |
| 167 | PRDX3 | na |  |  | 17543 | -1.275 | -0.1907 | Yes |
| 168 | LSM2 | na |  |  | 17575 | -1.284 | -0.1839 | Yes |
| 169 | VDAC1 | na |  |  | 17636 | -1.308 | -0.1784 | Yes |
| 170 | UBE2L3 | na |  |  | 17758 | -1.362 | -0.1758 | Yes |
| 171 | HDAC2 | na |  |  | 17939 | -1.461 | -0.1756 | Yes |
| 172 | RPL14 | na |  |  | 18032 | -1.501 | -0.1706 | Yes |
| 173 | SNRPD3 | na |  |  | 18050 | -1.511 | -0.1615 | Yes |
| 174 | APEX1 | na |  |  | 18097 | -1.547 | -0.1537 | Yes |
| 175 | AP3S1 | na |  |  | 18338 | -1.703 | -0.1551 | Yes |
| 176 | YWHAQ | na |  |  | 18355 | -1.715 | -0.1446 | Yes |
| 177 | EEF1B2 | na |  |  | 18369 | -1.728 | -0.1339 | Yes |
| 178 | SNRPB2 | na |  |  | 18409 | -1.762 | -0.1243 | Yes |
| 179 | HNRNPD | na |  |  | 18418 | -1.769 | -0.1131 | Yes |
| 180 | HPRT1 | na |  |  | 18443 | -1.794 | -0.1025 | Yes |
| 181 | RPS10 | na |  |  | 18522 | -1.855 | -0.0944 | Yes |
| 182 | YWHAE | na |  |  | 18585 | -1.908 | -0.0851 | Yes |
| 183 | NDUFAB1 | na |  |  | 18589 | -1.910 | -0.0726 | Yes |
| 184 | RPL22 | na |  |  | 18884 | -2.329 | -0.0727 | Yes |
| 185 | COX5A | na |  |  | 18911 | -2.373 | -0.0584 | Yes |
| 186 | RPL34 | na |  |  | 18950 | -2.457 | -0.0442 | Yes |
| 187 | UBE2E1 | na |  |  | 18984 | -2.546 | -0.0292 | Yes |
| 188 | ERH | na |  |  | 19067 | -2.796 | -0.0150 | Yes |
| 189 | PGK1 | na |  |  | 19154 | -3.315 | 0.0023 | Yes |
Table: GSEA details [plain text format]

  

Fig 2: HALLMARK\_MYC\_TARGETS\_V1      
 Blue-Pink O' Gram in the Space of the Analyzed GeneSet

  

Fig 3: HALLMARK\_MYC\_TARGETS\_V1: Random ES distribution      
 Gene set null distribution of ES for **HALLMARK\_MYC\_TARGETS\_V1**
